# Mental Disorders and Intimate Partner Femicide: Clinical Characteristics in Perpetrators of Intimate Partner Femicide and Male-to-Male Homicide

**DOI:** 10.3389/fpsyt.2022.844807

**Published:** 2022-03-21

**Authors:** Shilan Caman, Joakim Sturup, Katarina Howner

**Affiliations:** ^1^Department of Clinical Neuroscience, Centre for Psychiatry Research, Karolinska Institutet, Stockholm, Sweden; ^2^The Swedish Police Authority, Police Region Stockholm, Investigations Division, Stockholm, Sweden; ^3^Department of Forensic Psychiatry, National Board of Forensic Medicine, Stockholm, Sweden

**Keywords:** homicide, femicide, violence against women, intimate partner violence, offender, psychopathology, forensic psychiatry

## Abstract

Intimate partner violence against women is a global and persistent public health issue. An extreme manifestation of this problem is intimate partner femicide (IPF), the killing of a woman by a male partner. While declining trends of homicide rates have been observed over decades, rates of femicide and IPF have remained stable. Yet, IPF as a phenomenon has until recently been fairly invisible in Europe, why research from the European countries on rates and characteristics of IPF has been relatively scarce. One area of research, particularly in need of further scrutiny, is to what degree perpetrators of IPF suffer from mental health conditions, and what the clinical features are. The objective of present study was to add to the existing literature by investigating prevalence and types of mental disorders in perpetrators of IPF, and to compare with male-to-male homicide (MMH) perpetrators. Our aim was also to examine life-time contact with psychiatric services, and, with missed opportunities in mind, contacts shortly preceding the homicide. With a retrospective design, this population-based study includes all solved cases of male-perpetrated homicides against intimate female partners (IPF) and other males (MMH) committed in Sweden between January 2007 and December 2009. Primary and secondary psychiatric diagnoses based on ICD, version 8, 9 or 10 from psychiatric inpatient as well as outpatient care have been retrieved. In order to identify mental disorders in perpetrators during commission of the homicidal offense, we also retrieved diagnoses from forensic psychiatric evaluations. Our results demonstrate that approximately one-third of the perpetrators, irrespective of homicide type, had been diagnosed with a mental disorder (excluding substance related disorders) at some point in life. Diagnosis of substance related disorders from psychiatric care was significantly more common in MMH perpetrators (37%) compared to IPF perpetrators (15%). Similarly low rates of major mental disorder were found in both groups (11%) when aggregating life-time diagnoses and diagnoses during commission of the crime. However, homicide-suicide in connection to the offense was relatively common in IPF perpetrators (20%). Thus, our study supports the notion that previous suicide attempts and suicide ideation are important indicators for predicting and possibly preventing IPF.

## Introduction

Violence against women perpetrated by intimate partners is a global and persistent public health issue, with detrimental short- and long-term consequences. It has been estimated that approximately one in four women worldwide have been exposed to intimate partner violence (IPV) (1). While Sweden continually has been positioned as one of the leading countries in Europe (2) and globally (3) in terms of gender parity, IPV still constitutes an urgent and extensive societal issue. In line with the global estimates, 25% of women aged 15 years and older in Sweden have been victimized of IPV (4). According to Swedish crime statistics, approximately one in five of all assaults that were reported to authorities in 2019 involved victimization by a current or former partner, in which 84% of these cases involved a female victim (5). An extreme manifestation of violence against women is intimate partner femicide (IPF), the killing of a female by a male intimate partner. A recent report from the Centers for Disease Control and Prevention, based on homicides in 18 states, highlighted that 55% of the homicides committed against women in the U.S. involved an intimate partner (6). Moreover, roughly 60% of all female homicide victims are killed by their intimate (opposite-sex) partners, while the corresponding figure for men is below 10% in Sweden (7) and Europe (8), respectively. As such, the phenomenon of homicides against intimate partners is a gendered crime.

IPF has until recently been a fairly invisible phenomenon in the European research field, why research from European countries on this topic has been relatively scarce (9). As such, research on trends in rates of IPF has been fairly limited (10). One of several reasons for this has been the insufficient data regarding offender-victim relationships (11). Thus, the European Institute for Gender Equality emphasize the importance of data collection on femicides by EU Member States, in which, for example, information on background demographics, the context of the killing and the offender-victim relationship is included (2). On a similar note, the United Nations Office on Drugs and Crime (UNODC) report that the global data on gender-related killings of women and girls is of insufficient quality, leading to challenges in understanding the scale of the problem and monitoring trends (12). Nevertheless, the current available research demonstrates that rates of IPF tend to remain relatively stable over time in comparison to the overall homicide rates (9, 10, 13, 14).

The causes to intimate partner violence and IPF are complex; factors that increase or decrease risk appear on multiple levels and interplay (9, 15, 16). As demonstrated in Bronfenbrenner's Social Ecological System (17), which was first developed to study child development, relevant factors range from macro- to individual level. On macrolevel, factors such as gender inequality, public awareness and legislation may have an impact on intimate partner violence and IPF. On a community level, access to services, such as domestic violence resources, and norms within the community that support violence against women can contribute to the levels of risk. Factors at the interpersonal level include state and status of the intimate relationship, poverty and other family influences, such as child custody matters or presence of a stepchild. Lastly, there are individual-level risk factors, that is an individual's biological or personal history that influence the risk of becoming a perpetrator or victim. These factors, for example, relate to individual attitudes, mental health, substance abuse, and history of violence (9, 18). The focus in the present study is on individual-level factors in perpetrators, related to mental and substance use disorders.

Identification of risk factors specific for IPF is of great importance, as it enables the possibility to predict and to identify individuals at risk of greatest harm. Factors pertaining to different forms of previous IPV have been identified as the strongest risk factors for IPF (19–21). For example, the study on risk factors for femicide in abusive relationships by Campbell et al. (19) identified previous threats with a gun, stalking, forced sex and abuse as significant risk factors for IPF on bivariate-level. On a similar note, a recent meta-analysis identified history of non-fatal strangulation, previous rape of the victim, controlling behaviors, threat with a weapon, and previous threats to harm the victim as risk factors for IPF (21). All which tap in to different forms of previous IPV. In addition, direct access to a gun has been identified to be an independent and strong risk factor for IPF (19, 21). A cross-national study, involving data from 15 nations, indicates a five-fold higher risk of IPH (regardless of gender) when the perpetrator has direct access to a gun (22). However, a recent study from Spain did not identify previous reports of IPV or gun threats as independent risk factors for IPF (23). Moreover, men who kill intimate partners tend to be particularly possessive; demonstrated by controlling behaviors, jealousy and stalking (23–26), and a high proportion of IPFs are motivated by separation and/or jealousy (27). Separation, involuntary for the perpetrator, has been identified as a circumstance that elevates risk for IPF (24–26), and the level of risk is especially increased if the victim has a new partner (19). It is however important to keep in mind that women may leave as a response to IPV that escalates to a dangerous level (28).

Overall, factors related to perpetrators are particularly important when assessing risk for IPF (20). Unemployment has been identified as one of few risk factors related to sociodemographic background (29, 30). On a similar note, the perpetrator having economic or work-related problems in the past 6 months has been identified as one of the most important risk factors in a recent study (23). It has also been emphasized that suicide ideation and suicidal thoughts ought to be considered important in terms of risk for IPF (13, 23, 31), and it has been found that the suicide rate is four times higher in IPF perpetrators compared to perpetrators of other homicide types (32).

Mental disorders and substance use disorders have been identified to be risk factors for future IPV and IPF perpetration (19, 23, 33–35). However, the literature is somewhat unclear in what types of mental disorders actually pose an increased risk of severe or lethal violence against an intimate partner. Overall, aspects concerning clinical features in IPF perpetrators are under-researched and in particular need of further scrutiny (29).

Findings from a meta-analysis on risk factors for male IPF perpetration suggest that substance abuse by perpetrators significantly increase the risk of IPF, while history of mental health issues was found to be a significant but weaker risk factor (21). A consecutive case series of all intimate partner homicides and other adult domestic homicides in England and Wales between 1997 and 2008 reported that approximately one-third of all intimate partner homicide perpetrators (including both genders) had a lifetime diagnosis of a mental disorder, and the most common diagnosis was affective disorder (36). In terms of symptoms of mental disorder at the time of the intimate partner homicide, it was reported that 20% of the perpetrators had symptoms of mental disorders, 13% involved symptoms of depression and 7% involved symptoms of psychosis (36). In contrast, findings from a Swedish population-based study on homicides committed in Sweden between 1990 and 1999 concluded that a profound majority of the IPF perpetrators were mentally disordered, and that every third offender was psychotic at the time of the offense (32). Another register-based case-control study, involving men who had killed intimate partners with whom they had biological children, investigated psychiatric and criminal risk factors, and risk estimates relative to matched population controls (37). Major mental disorder, in their study defined as psychotic, personality and affective disorders, was found to be a strong independent risk factor, in which affective disorders were predominant (37).

Perpetrators of IPF have been suggested to be positioned in the middle of a psychopathological continuum; perpetrators of domestic homicides that involve non-intimates are more likely to exhibit psychopathological traits, while perpetrators of homicides involving acquaintances and strangers are less likely to be characterized with such attributes (27, 36, 38). However, the empirical findings regarding comparisons between IPF perpetrators and other homicides perpetrators are inconsistent. While some studies either demonstrate that perpetrators of IPF had been equally troubled (39) or more troubled (40) with regards to history of mental disorders, other findings even demonstrate that IPF perpetrators are *less* mentally troubled in some aspects. For example, a nationwide Finnish study reported that psychiatric contact before 18 years of age; antisocial personality disorder and drug abuse decreased the odds for IPF, as did psychoses and being assessed as legally insane (41).

Overall, studies on clinical features involving IPF perpetrators are relatively few and inconsistent. The reported prevalence rates of mental disorders vary between 10% (42) and 80% (32). We intended to address this gap of knowledge by investigating prevalence rates and types of mental disorders in perpetrators of IPF, and compare with male-to-male homicides (MMH). Our aim was to detect both longstanding illness and sudden onset of disorders. Doing so, we examined both diagnosis at some point in life prior and in connection to the offense. We also examined whether they had recent contact with psychiatric services, with missed opportunities in mind.

## Methods

### Sample Description

The present study is part of a larger project, in which a database called Forensic Homicide Database was manually created, incorporating all homicides in Sweden within a limited time frame (January 1st, 2007, through December 31st, 2009) [e.g., (7, 43–45)]. The nationwide and retrospective dataset is based on comprehensive and extensive information from various sources, that has been manually linked and systematically coded. Three national registries and police files were used to extract data on perpetrators and victims. In Sweden, a unique personal identification number is provided to all Swedish citizens, which enables linkage of registries. A forensic medicine registry by the National Board of Forensic Medicine provided data on victims who had died as a result of homicide. Victims and perpetrators were manually linked based on the police and court files, which also revealed the victim-perpetrator relationships. The database includes sufficient number of incidents in order to detect generalizable patterns and subtypes, however limited enough to be manageable and enable high-resolution details in which the complexity can be captured.

The adopted definition of homicide is in line with European research (46) and corresponds to intentional criminal acts of violence by one or more human beings resulting in the death of one or more other human beings. The definition holds cases of murder, manslaughter, infanticide, and finally aggravated assault/robbery *in combination with* causing another person's death. In terms of defining IPF, the present study refers to homicide against women in heterosexual relationships, in which the couples were or previously had been married, engaged, cohabitants or boyfriend-girlfriend.

The clearance rate for homicide incidents in Sweden during the study time was 87%, where a solved case denotes incidents that involve a perpetrator who has been charged or convicted, or where a prosecutor has identified the perpetrator(s) who could not be charged (for example if perpetrator has committed suicide or has gone missing). The unsolved cases of homicide consisted of 17% female victims and 83% male victims. For the purpose of present study, male-perpetrated homicides against intimate partners (i.e., IPF) were included, and compared to male-perpetrated homicides against other males. As such, male-perpetrated homicides against non-intimate females were omitted from the analyses, which is in line with previous similar studies (47, 48). Overall, the nationwide sample consist of 179 incidents of male-perpetrated homicides; 46 cases (26%) of IPF and 133 (74%) cases involving MMH. IPF perpetrators were significantly older (*Mdn* = 43 years) than perpetrators of MMH (*Mdn* = 29 years), U = 1,442, *p* < 0.001. In terms of country of birth, 59 % (*n* = 27) of IPF perpetrators and 68 % (*n* = 90) of MMH perpetrators were born in Sweden χ^2^, (1, *N* = 179) = 1.2, *p* = 0.270 (for a more detailed sample description see (43)).

### Measures

In order to identify psychiatric diagnoses prior to the index crime, the National Patient Register (NPR) from the National Board of Health and Welfare was used. The psychiatric inpatient registry provides all primary and secondary discharge diagnoses, with a nationwide mandatory documentation and county participation since 1987, and the psychiatric outpatient registry holds primary and secondary outpatient diagnoses, in which nationwide coverage was reached in 2001. The retrieved data from NPR consisted of information regarding dates (including admission and discharge dates for inpatient care) and diagnosis. The psychiatric diagnoses are coded according to the 8th, 9th, and 10th editions of the International Statistical Classification of Diseases and Related Health Problems (ICD; 1969e1986, 1987e1996, 1997e). Information was available on individuals treated both in psychiatric inpatient and outpatient facilities, in which some had been treated in both settings.

The National Board of Forensic Medicine is a governmental authority under the Ministry of Justice that is responsible for performing forensic psychiatric evaluations (FPE), requested by the courts to assess whether the offender suffered from a Severe Mental Disorder^1^ (SMD) in commission of the offense. During a pre-trial process, if it is suspected that the perpetrator may have suffered from a SMD at the time of the offense it is mandatory for the perpetrator to be subjected to an FPE. Additionally, with regard to severe crimes, such as homicide offenses, the court is more inclined to require an FPE. Firstly, the perpetrator is referred to a minor FPE (also called a §7-assessment in Sweden), an hour-long clinical assessment conducted by a specialist in psychiatry, with the objective of screening for indications of whether the perpetrator committed the crime under the influence of a SMD, and whether there is a need to continue with a major FPE. An FPE is conducted by a multidisciplinary team (forensic psychiatrist, clinical psychologist, social investigator and ward staff). When the suspected perpetrator is in custody the FPE is performed during 4 weeks on average and the perpetrator is admitted to a specific evaluation unit as an inpatient. The FPEs are based on observations, extensive interviews and retrospective records. The final assessment gives recommendations to the court whether the perpetrator ought to be sentenced to prison or compulsory forensic psychiatric care.

In order to identify presence of mental disorders in commission of the offense, primary and secondary diagnoses were retrieved for all perpetrators who underwent a major FPE. In total, 52% (*n* = 24) of the IPF perpetrators and 53% (*n* = 71) of the MMH perpetrators were subjected to a comprehensive FPE. As there were perpetrators who committed suicide in connection to the homicide offense, some perpetrators could not be subjected to an FPE.^2^ The diagnoses from the major FPEs were assessed according to the 4th text-revised edition of the Diagnostic and Statistical Manual of Mental Disorders (49), however; the registry retrieved was transformed and displayed according to the 10th version of ICD (50). Major mental disorder is defined as schizophrenia, schizoaffective disorder, bipolar disorder and/or depression with psychotic symptoms. Other diagnoses noticed were depression and anxiety, neuropsychiatric disorders including autism and ADHD, and other diagnoses (for example dementia, paraphilia, conduct disorder, stress reactions, adjustment disorders, Tourette syndrome, PTSD and intellectual disability). We also noticed personality disorders and substance abuse. The study was approved by the Regional Ethical Review Board in Stockholm, Sweden (Protocol-ID: 2010/1764–31/5).

### Statistical Analyses

The study has a descriptive and explorative approach, in which the Pearson's chi-square tests and, in variables with expected counts less than five, the Fisher exact tests were conducted for the categorical variables. In order to test the distribution of the continuous variable age, the Shapiro-Wilk test of normality was conducted, indicating deviation from normality. As such, Mann-Whitney U test was applied to analyze the age variable. Uncorrected probability values < 0.05, derived from two-tailed tests were regarded as statistically significant. Odds ratios (OR) were reported for categorical variables. A binary logistic regression was conducted in order to investigate timing of psychiatric inpatient and outpatient care. SPSS for Mac version 28 was used for all analyses.

## Results

### Previous Contacts With Psychiatric Services

As illustrated in Table 1, 41% (*n* = 19) of IPF and 53% (*n* = 70) of MMH perpetrators had received inpatient and/or outpatient care from psychiatric services prior to the homicide offending, χ^2^, (1, *N* = 179) = 1.8, *p* = 0.185. It is also worth mentioning that 33% (*n* = 15) of IPF perpetrators and 41% [*n* = 54; χ^2^, (1, *N* = 179) = 0.9, *p* = 0.708] of MMH perpetrators had received inpatient psychiatric care. The time aspect, in which how recent the contacts with the psychiatric services were, is interesting from a point of view of missed opportunities of intervention. A somewhat higher (non-significant) percentage of MMH perpetrators (28%, *n* = 37) had consumed psychiatric care during the past year, compared to IPF perpetrators [17%, *n* = 8; χ^2^, (1, *N* = 179) = 2.0, *p* = 0.160]. However, looking in to psychiatric care in closer proximity to the homicide offense, our results demonstrate that 11% (*n* = 5) of IPF perpetrators and only 6% (*n* = 8) of MMH perpetrators had sought psychiatric service the same month as the offense (*p* = 0.323). With an even higher resolution, our results demonstrate that the corresponding figures for contact with psychiatric services the same week as the offense are 7% (*n* = 3) in IPF perpetrators and 3% (*n* = 4) in MMH perpetrators (*p* = 375). Furthermore, two IPF perpetrators had been in contact with psychiatric services the same day as the homicide (related to dementia, respectively, substance use disorder). Results from a logistic regression (see Table 2) confirm these findings by showing a similar tendency. Contact with psychiatric services previously than or during the year of the offense were associated with lower odds for IPF, whereas contact during the month of the offense was associated with higher odds for IPF. However, this association was not statistically significant, conclusions should therefore be interpreted cautiously.

**Table 1:** Clinical characteristics in intimate partner femicide (IPF) and male-to-male homicide (MMH) perpetrators prior to the incident (national patient registry).

**Variable**	**IPF**	**MMH**	**χ^2^**	***p*** **-value**	**Odds ratio (OR)**	**95% confidence interval**
	***n*** **(%)** **46 (100)**	***n*** **(%)** **133 (100)**				
Any psychiatric care	19 (41.3)	70 (52.6)	1.8	0.185	0.633	(0.32–1.25)
Outpatient care	11 (23.9)	51 (38.3)	3.2	0.076	0.505	(0.24–1.08)
Inpatient care	15 (32.6)	54 (40.6)	0.9	0.337	0.708	(0.35–1.44)
Contact > year	11 (23.9)	33 (24.8)	0.02	0.903	0.952	(0.44–2.09)
**Contact** **<** **year**	**3 (6.5)**	**29 (21.8)**	**5.4**	**0.020**	**0.250**	**(0.07–0.87)**
Contact < month	5 (10.9)	8 (6.0)	F	0.323	1.905	(0.59–6.15)
Major mental disorder	2 (4.3)	11 (8.3)	F	0.520	0.504	(0.11–2.37)
Psychotic disorder	1 (2.2)	6 (4.5)	F	0.679	0.470	(0.06–4.01)
Bipolar and Schizoaffective disorder	1 (2.2)	5 (3.8)	F	1.000	0.569	(0.07–5.00)
Neuropsychiatric disorder	2 (4.3)	12 (9.0)	F	0.524	0.458	(0.10–2.13)
Depression/anxiety	8 (17.4)	22 (16.5)	0.02	0.894	1.062	(0.44–2.58)
Other diagnosis	7 (15.2)	24 (18.0)	0.2	0.662	0.815	(0.33–2.04)
Personality disorder	1 (2.2)	9 (6.8)	F	0.456	0.306	(0.04–2.49)
**Substance related disorder**	**7 (15.2)**	**49 (36.8)**	**7.4**	**0.006**	**0.308**	**(0.13**–**0.74)**
Any psychiatric diagnosis^*^	14 (30.4)	47 (35.3)	0.4	0.545	0.801	(0.39–1.65)
**Both psychiatric and substance related disorders**	**3 (6.5)**	**26 (19.5)**	**4.27**	**0.039**	**0.287**	**(0.83–0.99)**
**Homicide-suicide**	**9 (19.6)**	**2 (1.5)**	**F**	**<0.001**	**15.932**	**(3.30–76.97)**

* *Any psychiatric diagnosis including personality disorders excluding substance related disorders. Based on diagnoses from psychiatric outpatient and/or inpatient care*.

*The bold text indicates probability values less than .05 which are derived from two-tailed tests that were regarded as statistically significant*.

**Table 2:** Binary logistic regression regarding timing of psychiatric outpatient and inpatient care in intimate partner femicide (IPF) and male-to-male homicide perpetrators.

	***B***	**SE**	**Wald**	***P*** **-value**	**Odds ratio (OR)**	**95 % confidence interval for OR**
Contact > year	−0.251	0.417	0.363	0.547	0.778	(0.343–1.762)
Contact < year	−1.421	0.649	4.802	0.028	0.241	(0.068–0.861)
Contact < month	0.377	0.615	0.377	0.539	1.458	(0.437–4.866)
Nagelkerke R square = 0.059						

### Clinical Characteristics in Perpetrators

Diagnosis of a major mental disorder (i.e., schizophrenia, schizoaffective disorder, bipolar disorder and/or depression with psychotic symptoms) from psychiatric inpatient or outpatient care prior to the homicide offense was uncommon in IPF (4%, *n* = 2) and MMH perpetrators (8%, *n* = 11; *p* = 0.520). However, approximately every third perpetrator, irrespective of homicide type, had been diagnosed with a mental disorder prior to the homicide offense, in which substance related diagnoses had been excluded. Additionally, 15% (*n* = 7) of the IPF perpetrators had been diagnosed with a substance use disorder, which is significantly lower than in MMH perpetrators [34%, *n* = 49; χ^2^ (1, *N* = 179) = 7.4, *p* = 0.006]. Considering the combination of both psychiatric disorders and substance related disorders based on the NPR, our results demonstrate a significant difference between the two groups; 7% (*n* = 3) in IPF perpetrators and 20% (*n* = 26) in MMH perpetrators [χ^2^ (1, *N* = 179) = 4.3, *p* = 0.039].

With regard to major mental disorders in commission of the index crime, we investigated the psychiatric diagnoses in all IPF (*n* = 24) and MMH perpetrators (*n* = 71) who underwent a major FPE. Among these, four of the IPF perpetrators suffered from a major mental disorder during commission of the crime, in which two were related to psychosis. The corresponding figures in MMH perpetrators are eight and six. Overall, aggregating both life-time diagnoses (according to the NPR) and diagnoses during commission of the crime (according to the FPEs), it is demonstrated that major mental disorders was found in 11% (*n* = 5) of IPF perpetrators, and in 11% (*n* = 15) of MMH perpetrators: similarly low rates in both groups [χ^2^ (1, *N* = 179) = 0.006, *p* = 0.940]. With regards to personality structure, there were eight IPF perpetrators and 13 MMH perpetrators who were diagnosed with a personality disorder (predominantly borderline and antisocial personality disorders). The findings on combination of psychiatric disorders and substance related disorders, after aggregating data from NPR and FPEs, demonstrate fairly similar rates between IPF perpetrators (22%, *n* = 10) and MMH perpetrators [27%, *n* = 36; χ^2^, (1, *N* = 179) = 0.6, *p* = 0.476].

A result differentiating IPF and MMH perpetrators is with regards to homicide-suicide; 20% (*n* = 9) of the IPF perpetrators committed suicide within 24 h after the incident, which is significantly higher compared to MMH perpetrators (2%, *n* = 2; *p* < 0.001). Among the nine IPF perpetrators who committed suicide, five had been visiting a psychiatric health service prior to the offense, in which two had the preceding contact within the same month as the homicide offense.

## Discussion

Firstly, our objective was to contribute to the scientific literature regarding clinical characteristics in IPF perpetrators, by investigating the prevalence rates and types of mental disorders in IPF and MMH perpetrators, respectively. We were particularly interested in the prevalence rates of major mental disorders (i.e., psychoses, bipolar disorders, schizoaffective disorders or depressions with psychotic symptoms), both prior to and in connection to the homicide. Major mental disorders could be related to longstanding illness rather than sudden onset. Secondly, we intended to investigate the extent to which IPF and MMH perpetrators had preceding contact with psychiatric outpatient and inpatient services prior to the homicide. This approach helps to identify opportunities, or lack thereof, to prevent homicides in terms of perpetrators interacting with different authorities, such as the mental health services.

Our results demonstrate that ~40% of IPF perpetrators had received mental health care at some point in life, prior to the offense. With regards to psychiatric inpatient care, our results are nearly identical to the findings by Weizmann-Henelius et al. (41), in which it was found that 32% of IPF perpetrators had been committed to psychiatric inpatient care. Considering recent contact with mental health services, Oram et al. (36) found that 14% had been visiting a mental health service during the same year, and 9% during the same month as the homicide. These findings are relatively similar to our corresponding figures of 17 and 11%, respectively. An even higher resolution, illustrated that three IPF perpetrators had contact with the mental health services the same week, in which two of these were discharged from psychiatric inpatient care the same day. Thus, despite small number of cases, our findings indicate that there are, in fact, some opportunities of risk assessment and intervention. Our study also highlights a tendency related to timing of psychiatric care; any psychiatric care was somewhat less common in IPF perpetrators in comparison to MMH perpetrators, however, among those who had received any psychiatric care, IPF perpetrators tended to have more recent contact with the mental health services. This could perhaps indicate some kind of crisis or worsened psychiatric state in these perpetrators, which introduces opportunities of intervention. However, this association was not statistically significant and the results should be verified in other studies.

In light of clinical characteristics, the study by Oram et al. (36) demonstrated that approximately one-third of all intimate partner homicide perpetrators had a lifetime diagnosis of a mental disorder, excluding substance use, in which affective disorders were predominant. Similarly, Bridger et al. (13) found that one-third of IPF perpetrators had diagnosis of a mental disorder, mostly involving depression. These findings are supported by the results in our study; while there was a low rate of major mental disorders, we found that ~30% of IPF perpetrators had been diagnosed with any mental disorder (excluding substance use disorders) prior to the homicidal act, in which depression was predominant.

Previous comparable studies have highlighted that IPF and MMH perpetrators differ with regards to substance use disorders, in which IPF perpetrators are less likely to suffer from substance abuse (32, 39, 41). However, some research demonstrates high rates of chronic substance use disorders in IPF perpetrators (13). Based on perpetrators being diagnosed with a substance use disorder at a mental health service *prior* to the homicide offense, IPF perpetrators show significantly less adversity in this regard. However, when the data from the FPE was added, similar rates of substance use disorders were detected in IPF and MMH perpetrators. This could indicate that, rather than having less substance related issues, they may in fact be less likely to be diagnosed with substance use disorders, maybe due to not seeking help or the health care system not acknowledging the substance use in this group.

Considering personality disorders, it has been pointed out that overcontrolled-dependent men have been overlooked in terms of risk for IPF, and that the personality disorder most likely to be involved in IPF is men with dependent and passive-aggressive tendencies (51). In a similar vein, it has been concluded that psychopathic traits are rare in IPF perpetrators, and that they predominantly exhibit borderline/dysphoric traits (32). Considering the low tolerance for separation in borderline personality disorder (49), and involuntary estrangement being a common circumstance in these killing (19, 24), these findings may not be surprising. As such, the type of personality disorder could be a possible difference between IPF and MMH perpetrators, in which borderline personality disorder is more common in the IPF group, and antisocial personality disorder in the MMH group. However, the sample size was too small in order to allow satisfactory statical analyses between the groups. Nonetheless, our findings highlight existence of both borderline and antisocial personality disorders in IPF perpetrators. Moreover, our findings demonstrate that the frequencies of personality disorders in the FPEs were high; approximately one-third in both groups were diagnosed with a personality disorder, while they rarely had been diagnosed in outpatient and inpatient psychiatric care. This may suggest that personality disorders probably are underdiagnosed in psychiatric care, and don't necessarily lead to treatment. It also reflects the deeper investigation in the FPE.

Major mental disorders, in present study operationalized as psychoses, bipolar disorders, schizoaffective disorders or depressions with psychotic symptoms, were relatively uncommon, even after aggregating information life-time diagnoses (from the NPR) and diagnoses during commission of the offense (from the FPEs). In total, 11% of homicide perpetrators, regardless of homicide type, had been diagnosed with a major mental disorder, prior or in connection to the offense. This percentage is considerably lower than the results found by Belfrage and Rying (32) demonstrating that every third IPF perpetrator is psychotic at the time of the offense, however, more in line with the findings by Oram et al. (36), indicating that 7% had suffered from psychosis. The inconsistencies between findings in current study and the findings elucidated by Belfrage and Rying (32) are probably explained by how the psychotic disorders were defined, as the latter study included all depressions in the definition of psychoses. Overall, statistics regarding mental disorders in homicide offenders vary significantly between studies, and there can be variations in diagnostic methods, making it challenging to obtain accurate prevalence rates (52). Another possible reason for the conflicting evidence may concern inconsistent definitions and insufficient operationalization of mental disorders, which hinders comparisons across studies (29, 53). A wide range of concepts have been used regarding mental conditions (e.g., mental disorder, mental illness, major mental disorder, and severe mental illness), and sometimes same concepts have been used for different conditions. Transparency in operationalizations of these concepts is therefore key, as is disaggregated presentation of the diagnoses since it enables comparisons across studies.

The rates of homicide-suicide have remained stable over time (12, 54), and are low in comparison to overall homicide and suicide rates, respectively (55). As has been found in previous research (32, 56), our findings highlight that the phenomenon of homicide-suicide is closely related to IPF; while one in five IPF perpetrators committed suicide in connection to the offense, suicide among MMH perpetrators was rare. As such, our study supports the notion that previous suicide attempts and suicide ideation are important indicators for predicting and possibly preventing IPF (23). The importance of mental disorders in homicide-suicides has been emphasized in previous research (57, 58). For example, in a sample of homicides committed in Spain, mental disorders were found to be four times more common in homicide-suicide perpetrators, compared to perpetrators of general homicides (55). As such, it has been theorized that the combination of mental disorders and a stressful event, like separation, is a plausible explanation for homicide-suicides in cases of IPF (55). It is, however, worth mentioning that different types of homicide-suicides have been identified; one type that is predominantly driven by homicidal intention, where the suicide is motivated by avoiding legal and social consequences, while the other type predominantly is related to suicidal intent, in which the homicide is an extension of the suicide (23, 59).

### Implications for Practice and Future Research

In national homicide death reviews, so called fatality reviews, possible missed opportunities of intervention and system gaps are identified and analyzed. Fatality reviews in some countries also identify risk factors, which may provide valuable information beyond prediction of repeated IPV (60), and shed light on possible risk factors unique for lethal violence. The aim of fatality reviews is to prevent future homicides by providing recommendations for improved practices, procedures and systems (9, 18). An annual report from the Domestic Violence Death Review Committee in Ontario, Canada (60) recommends that professionals within mental health services and addiction care receive training on risk factors for intimate partner femicide. They also encourage that presence of risk factors, such as depression and access to firearms, should lead to risk assessments, risk management and safety planning (60). The relevance of these recommendations is corroborated by the findings in present study, in which it has been demonstrated that a group of IPF perpetrators had recent contact with the mental health services prior to the offense. A study on intimate partner homicides in Norway, based on court documents and interviews with bereaved, illustrates that when individuals conveyed IPV related concerns to professional agencies, there was a tendency by professionals to not comprehend the urgency and level of risk, and did therefor not act on these reports (54). Moreover, a recent fatality review from Sweden (61) shows that even when the level of risk is comprehended, no contact is initiated with the law enforcement. Thus, it is of great importance that mental health professionals, as well as social service providers, inform the police in cases of potential danger, in which one should pay special attention to depressive and suicidal tendencies. Previous research has also shown that police officers have particular difficulty assessing aspects related to mental disorders in suspects of IPV (62), which suggests that training for professionals within the law enforcement regarding risk factors is warranted.

Given that clinical characteristics of mental disorders, substance use, and suicide are risk factor for IPF, and relatively prevalent among IPF perpetrators, accessible care and services targeting these issues may have a preventive effect. However, one identified system gap is that sufficient treatment for substance use and mental disorders had not been provided to perpetrators of IPF prior to the killing (61). In general, the health care system is an important piece of the puzzle in terms of prevention and intervention opportunities to combat IPV and IPF.

Risk assessment and management are critical components with regards to preventing IPV recidivism and IPF (23). There are, however, challenges related to prediction of IPF. For example, there are studies indicating differences between IPV and IPF (19, 24), in which IPF is considered more complex (23). Yet, most risk assessments have a global predictive target, intended to assess risk for IPV recidivism (23). Furthermore, since rare events are more difficult to predict, risk assessment tools targeting IPF have lower predictive validity (63). Scholars have therefore recommended using two complementary risk assessment in order to increase the predictive capacity (64). Another important challenge worth highlighting is the lack of a single type of IPF perpetrator (23). Previous research indicates heterogeneity among IPF perpetrators (27), and who may display different risk indicators. In a similar vein, Dawson and Piscitelli (65) emphasize that future research on IPF risk factors ought to investigate certain combination of risk factors (i.e., clusters) instead of regarding these as independent of one another. For example, previous research demonstrates that the combination of psychiatric disorders and substance use disorders give the highest risk for violence (66). In present study, 22% of IPF perpetrators and 27% of MMH perpetrators displayed comorbidity of both psychiatric and substance use disorders. This new approach to risk factors, in which the combination of factors is considered, may improve risk assessment and management.

### Limitations

The present study is not without limitations. First and foremost, the relatively low rates of homicide in Sweden and, furthermore, the low rates of mental disorders within these subgroups, makes it challenging to sufficiently identify differences with regards to mental disorders and to use satisfactory statistical methods based on the limited time-frame adopted in present study. For example, one would preferably use a multiple logistic regression, in order to control for confounders. An additional limitation related to the time-frame is that the data is based on a sample from previous years. However, except for the extraordinary circumstances related to the Covid-19 pandemic, the rates and characteristic of IPF tend to be relatively stable over time (10), why the present findings ought to be of current relevance. For example, the overall rates of IPF have remained relatively stable in Sweden since the early 1990s, demonstrating a modest decline. Also, the majority of the characteristics in IPF perpetrators, such as ethnicity and criminal history, have remained stable over time (10). Furthermore, in spite of the obstacles related to statistical methodology, present study identifies descriptive data on approximately how common, or rare, various mental disorders are in a representative sample of perpetrators. On the other hand, the study would have been improved if diagnostic data from the primary health care was included, rather than restricting the data to diagnoses from mental health care, and would reasonably provide higher estimates. Furthermore, as the psychiatric outpatient registry is not as complete as the inpatient registry, there is a risk of underestimating the prevalence of mental disorders or exposure to mental health care. By using both inpatient and outpatient data, we optimize the chances of identifying these aspects. An additional limitation of the study is the lack of matched controls, facilitating comparisons to population figures in terms of prevalence rates of mental disorders.

It is also worth mentioning that a small proportion of the unsolved cases involved femicide (17%), and a previous study based on the current dataset has demonstrated that the unsolved cases predominantly involve young men who are criminally active (45). A methodological advantage of present study is the representative sample, since all perpetrators are convicted of their crimes in Sweden, regardless of their mental state during commission of the offense. This is especially fundamental when aiming to investigate mental disorders in these perpetrators, since the individuals who suffer from major mental disorders might be less likely to become convicted in some countries, and therefore tend to not be included in some datasets. Furthermore, present study includes homicide-suicide cases, which additionally improves the representativeness of the sample, considering the fact that a substantial proportion of IPF perpetrators commit suicide, and are important to include. On the other hand, this subgroup was not represented with regards to mental status in connection to the offense, as perpetrators who committed suicide in connection to the offense could not be subjected to FPEs. Although no conclusions can be drawn with regards to this, it is reasonable to assume that this subgroup is characterized by adversities related to mental disorders, such as depression and crisis reactions.

### Conclusions

Unraveling IPF perpetrators use of psychiatric services, and their clinical characteristics, can be essential for identification of high-risk individuals, and for understanding the prospects of efficient intervention. Our study indicates that there are possible opportunities of risk assessment and intervention, as some IPF perpetrators had recent contact with the mental health services prior to the offense. Overall, approximately one-third of all perpetrators had been diagnosed with a mental disorder at some point in life prior to the homicide, while only a minority of IPF perpetrators displayed characteristics of major mental disorders. On the other hand, homicide-suicide in connection to the offense was relatively common in IPF perpetrators. As such, our study supports the notion that previous suicide attempts and suicide ideation are important indicators for predicting and possibly preventing IPF.

## Data Availability Statement

The raw data supporting the conclusions of this article will be made available by the authors, without undue reservation.

## Ethics Statement

The studies involving human participants were reviewed and approved by Regional Ethical Review Board in Stockholm, Sweden (Protocol-ID: 2010/1764–31/5). Written informed consent for participation was not required for this study in accordance with the national legislation and the institutional requirements.

## Author Contributions

JS collected and linked all data and all authors (JS, SC, and KH) contributed in coding the data. SC conducted the statistical analyses and wrote the manuscript. All authors contributed in conceptualizing and operationalizing the study aim and approach, development and revision of the manuscript, and read and approved the submitted version.

## Conflict of Interest

The authors declare that the research was conducted in the absence of any commercial or financial relationships that could be construed as a potential conflict of interest.

## Publisher's Note

All claims expressed in this article are solely those of the authors and do not necessarily represent those of their affiliated organizations, or those of the publisher, the editors and the reviewers. Any product that may be evaluated in this article, or claim that may be made by its manufacturer, is not guaranteed or endorsed by the publisher.

## Footnotes

^1^Severe Mental Disorder is a judicial term that holds (1) all psychotic states regardless of origin, (2) severe depression with suicidal ideation, (3) personality disorders with psychotic episodes, (4) mental disorders with marked compulsiveness with an impact on the social functioning, and (5) severe intellectual disability, severe dementia and severe brain damage.

^2^Taken this into account, the figures in which the homicide-suicide perpetrators have been excluded are 65% (24/37) of IPF perpetrators and 54% (71/131) of MMH perpetrators.
